# Three AtCesA6‐like members enhance biomass production by distinctively promoting cell growth in *Arabidopsis*

**DOI:** 10.1111/pbi.12842

**Published:** 2017-10-23

**Authors:** Huizhen Hu, Ran Zhang, Shengqiu Feng, Youmei Wang, Yanting Wang, Chunfen Fan, Ying Li, Zengyu Liu, René Schneider, Tao Xia, Shi‐You Ding, Staffan Persson, Liangcai Peng

**Affiliations:** ^1^ Biomass and Bioenergy Research Centre Huazhong Agricultural University Wuhan China; ^2^ National Key Laboratory of Crop Genetic Improvement Huazhong Agricultural University Wuhan China; ^3^ College of Plant Science and Technology Huazhong Agricultural University Wuhan China; ^4^ Max‐Planck‐Institute of Molecular Plant Physiology Potsdam‐Golm Germany; ^5^ School of Biosciences University of Melbourne Parkville VIC Australia; ^6^ College of Life Science and Technology Huazhong Agricultural University Wuhan China; ^7^ Department of Plant Biology Michigan State University East Lansing MI USA

**Keywords:** cellulose synthesis, cell expansion, cell division, CesA, cell wall, biomass production

## Abstract

Cellulose is an abundant biopolymer and a prominent constituent of plant cell walls. Cellulose is also a central component to plant morphogenesis and contributes the bulk of a plant's biomass. While cellulose synthase (*CesA*) genes were identified over two decades ago, genetic manipulation of this family to enhance cellulose production has remained difficult. In this study, we show that increasing the expression levels of the three primary cell wall *AtCesA6*‐like genes (*AtCesA2*,* AtCesA5*,* AtCesA6*), but not *AtCesA3*,* AtCesA9* or secondary cell wall *AtCesA7*, can promote the expression of major primary wall *CesA* genes to accelerate primary wall CesA complex (cellulose synthase complexes, CSCs) particle movement for acquiring long microfibrils and consequently increasing cellulose production in *Arabidopsis* transgenic lines, as compared with wild‐type. The overexpression transgenic lines displayed changes in expression of genes related to cell growth and proliferation, perhaps explaining the enhanced growth of the transgenic seedlings. Notably, overexpression of the three *AtCesA6*‐like genes also enhanced secondary cell wall deposition that led to improved mechanical strength and higher biomass production in transgenic mature plants. Hence, we propose that overexpression of certain *AtCesA* genes can provide a biotechnological approach to increase cellulose synthesis and biomass accumulation in transgenic plants.

## Introduction

Plant cell walls provide the raw material for a range of important industries, including feed, food, fuel and materials (Carroll and Somerville, 2009; Somerville, 2006). The cell wall is also essential for plant growth and development as it determines plant cell size and shape and provides structural growth support and protection against various environmental stresses (Landrein and Hamant, 2013; Le Gall *et al*., 2015; Malinovsky *et al*., 2014; Szymanski and Cosgrove, 2009). Plant cell walls are comprised largely of polysaccharides (cellulose, hemicellulose, pectin) and the polyphenolic structure lignin (Somerville *et al*., 2004). In general, two major types of plant cell walls are characterized: a thin, pectin‐rich primary cell wall surrounds all dividing and expanding cells; a thickened lignin‐rich secondary cell wall provides structural support to specialized cells, such as xylem cells (Harholt *et al*., 2010; Scheller and Ulvskov, 2010; Somerville *et al*., 2004; Wang *et al*., 2016a). In particular, primary wall synthesis is closely associated with cell division and expansion that determine the size of an organ/tissue, whereas secondary wall deposition is initiated during the process of cellular differentiation to contribute plant strength and overall biomass production (Keegstra, 2010; Schuetz *et al*., 2013).

Cellulose is composed of β‐1,4‐linked glucan chains that interact with one another via hydrogen bonds to form para‐crystalline microfibrils (Peng *et al*., 2002; Schneider *et al*., 2016; Somerville *et al*., 2004). As the most prominent and load‐bearing component of many plant cell walls, cellulose plays a central role in plant mechanical strength and morphogenesis (Cosgrove, 2005; Liu *et al*., 2016). In most land plants, cellulose is synthesized by a large cellulose synthase (CesA) complex at the plasma membrane (Schneider *et al*., 2016). In *Arabidopsis*, CesA1, CesA3 and one of four CesA6‐like proteins (CesA6, CesA2, CesA5 and CesA9) are involved in primary wall cellulose synthesis, whereas CesA4, CesA7 and CesA8 are essential isoforms for secondary wall cellulose synthesis (Desprez *et al*., 2007; McFarlane *et al*., 2014; Persson *et al*., 2007; Taylor *et al*., 2003). Furthermore, CesA1 and CesA3 are essential for plant growth because mutation of each gene leads to lethality (Persson *et al*., 2007). By comparison, mutations in any one of the *CesA6*‐like genes cause only mild growth phenotypes (Cano‐Delgado *et al*., 2003; Scheible *et al*., 2001). However, *cesa5 cesa6* double mutants are seedling lethal (Desprez *et al*., 2007), and *cesa2 cesa6 cesa9* triple mutants are gamete lethal, probably due to *CesA9* tissue‐specific floral expression (Persson *et al*., 2007). Because *CesA2* or *CesA5* expression driven by a *CesA6* promoter only partially complements *cesa6* mutant phenotypes (Desprez *et al*., 2007; Persson *et al*., 2007), it is assumed that each *CesA6*‐like gene may have obtained specialized functions.

Because cellulose is importance for plant biomass exploitation, increased production of this polymer in a plant is highly desirable (Burton and Fincher, 2014), and many efforts have been undertaken to increase cellulose synthesis. However, as CesA family genes were identified over two decades ago (Arioli *et al*., 1998; Pear *et al*., 1996), genetic manipulation of its members to enhance cellulose production has remained difficult. For instance, overexpression of *CesA* genes (mainly secondary wall *CesAs*) has not led to improved plant growth (Joshi *et al*., 2011; Li *et al*., 2017; Tan *et al*., 2015; Wang *et al*., 2016b). Here, we demonstrated that overexpression of any of the three *CesA6*‐like genes *CesA2*,* CesA5* or *CesA6* can increase cellulose production in *Arabidopsis*. The overexpressing transgenic lines showed increased cell expansion and division and also enhanced secondary cell wall deposition. Hence, alterations in the expression of certain primary wall *CesA* genes may offer avenues to enhance cellulose synthesis and biomass production in plants.

## Results

### Overexpression of three *CesA6‐*like genes enhances seedling growth in *Arabidopsis*

We used either dark‐grown (D) or light‐grown (L) seedlings to study how different aspects of plant development correlated with changes in cellulose synthesis (Figure S1a). We investigated in detail growth and transcript levels of the *CesA* genes associated with primary and secondary wall cellulose synthesis using real‐time PCR (Q‐PCR) analysis in *Arabidopsis* wild‐type (WT; Col‐0) seedlings (Figure S1b–e). Based on these data, we found a consistently enhanced growth at both 9‐day‐old dark‐grown (D9) hypocotyls and 9‐day‐old light‐grown (L9) roots and chose these tissues to measure primary cell wall deposition relating to cell length and cell numbers, due to their relatively large tissue size and high primary wall *CesA* expression levels.

**Figure 1:**
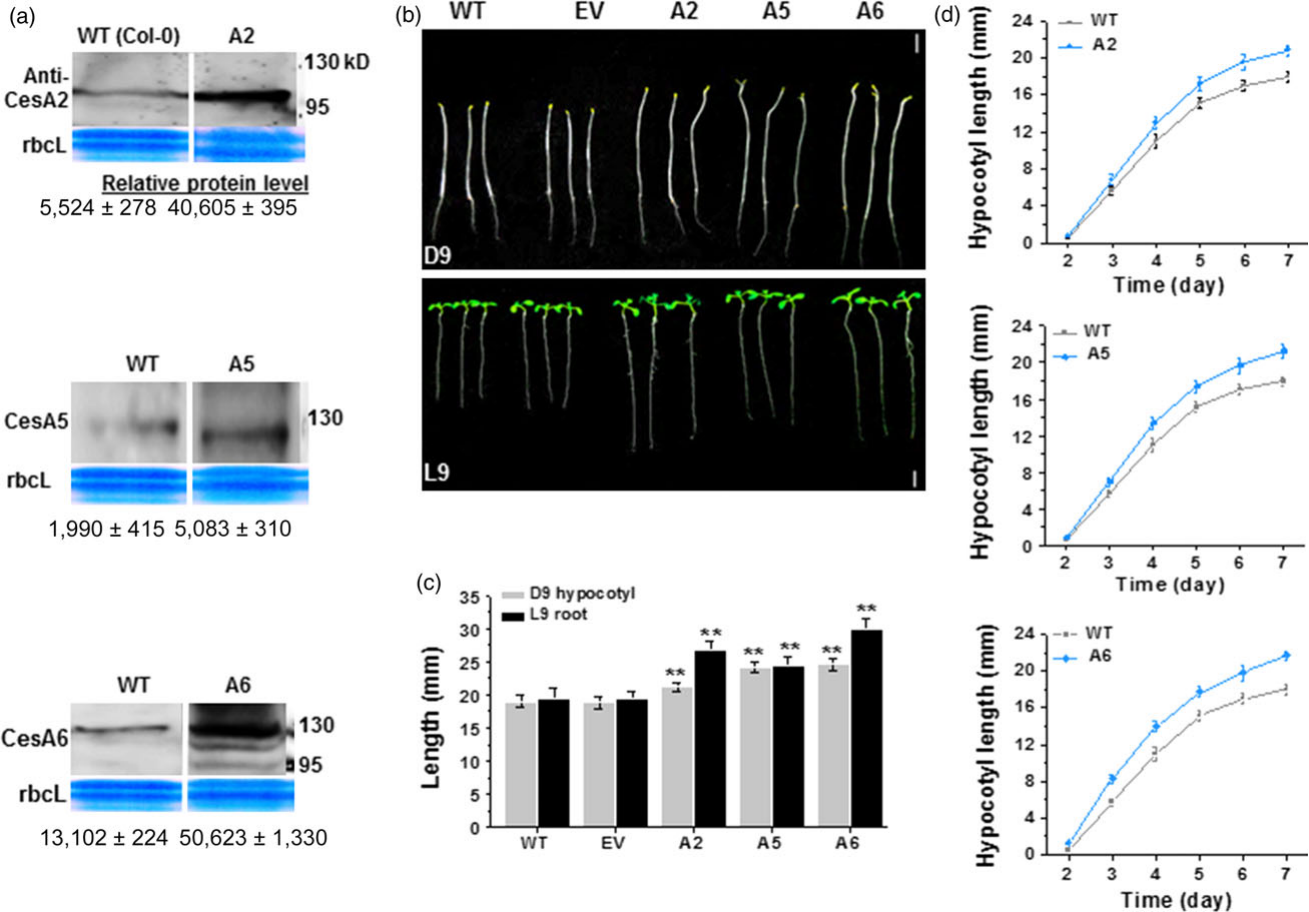
Enhanced seedling growth in three *CesA6‐*like overexpressing *Arabidopsis* plants. (a) Western blot analyses of CesA2, CesA5 and CesA6 proteins of D9 seedlings as shown in (b). Data indicated means ± SD; three WT lanes were derived from the same reference gel, and all blot analyses used the same amounts of protein samples. (b) Homozygous *Arabidopsis* seeds germinated and grown on 1/2 MS media for 9 days under dark (D9; 24 h dark) or light (L9; 16‐h light: 8‐h dark) conditions. WT as wild type (Col‐0); EV as transgenic plants transformed with empty vector; the A2, A5, A6 were the transgenic plants that overexpressed *CesA2*,* CesA5* and *CesA6* genes, respectively; Scale bars, 5 mm. (c) Hypocotyls and roots lengths as shown in (b). Bars indicated means ± SD (*n* = 3 biological replicates), and at least 50 seedlings were measured in each replicate; Student's *t*‐tests were performed between WT and transgenic plants as **P <* *0.01. (d) The growth curve of D2–7 hypocotyls in A2, A5 and A6 lines. Data indicated means ± SD (*n* = 3 biological replicates), and at least 30 seedlings were measured in each replicate.

To explore whether overexpression of certain *CesAs* may improve plant growth and cellulose synthesis, we generated *CesA* overexpressing lines driven by 35S promoter in *Arabidopsis* WT background. We subsequently monitored growth of the homozygous transgenic progeny. At least three genetically independent homozygous transgenic lines were selected for each gene, and the lines were verified by Western blot analysis of protein levels (Figures 1a and S2b–d). Interestingly, compared with WT and empty vector (EV) plants, transgenic lines overexpressing *CesA2* (A2), *CesA5* (A5) and *CesA6* (A6), but not *CesA3* (A3), *CesA9* (A9) and *CesA7* (A7), showed longer hypocotyl or root length (Figures 1b–d and S3). These data indicate that overexpression of certain *CesA6*‐like genes can enhance *Arabidopsis* seedling growth.

### Overexpression of three *CesA6‐*like genes increases the expression of other primary wall *CesAs*

**Figure 2:**
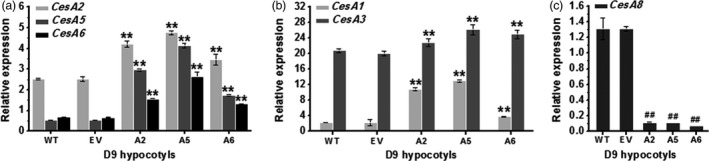
Q‐PCR analyses of *CesA* genes in D9 hypocotyls of three *CesA6‐*like genes overexpressing lines. (a) *CesA2*,* CesA5* or *CesA6* genes, (b) *CesA1* or *CesA3* genes and (c) *CesA8* gene. *GAPDH* was used as the internal control, and the expression value of *GAPDH* was defined as 100; bars indicated means ± SD (*n* = 3 biological replicates); Student's *t*‐tests were performed between WT and transgenic plants as **P <* *0.01 for increase or ^##^ *P *<* *0.01 for decrease.

To investigate how the enhanced seedling growth was supported in the overexpression lines (A2, A5 and A6), we first analysed the expression level of major *CesA* genes in young seedlings by Q‐PCR (Figures 2 and S4). We found that overexpression of one of the *CesA2*,* CesA5* and *CesA6* genes could enhance the others both in D9 hypocotyls (Figure 2a) and in L9 roots (Figure S4a). Notably, the other two major primary *CesA* genes (*CesA1* and *CesA3*) also typically displayed significantly increased expression levels, especially in D9 hypocotyls. While expressions of nearly all primary wall *CesAs* were increased in the transgenic lines, the *CesA3* expression was curiously reduced in seedling roots of the lines (Figures 2b and S4b). However, one of the major secondary wall CesAs, the *CesA8* gene, showed markedly decreased expression levels in both D9 hypocotyls and L9 roots (Figures 2c and S4c). Taken together, overexpression of any of the three *CesA6‐*like genes could increase the expression of other primary wall *CesAs* genes, with the exception of *CesA9*, which is mainly expressed in pollen tissues.

### Overexpression of three *CesA6‐*like genes increases dynamic movement of primary wall CesAs in the plasma membrane

**Figure 3:**
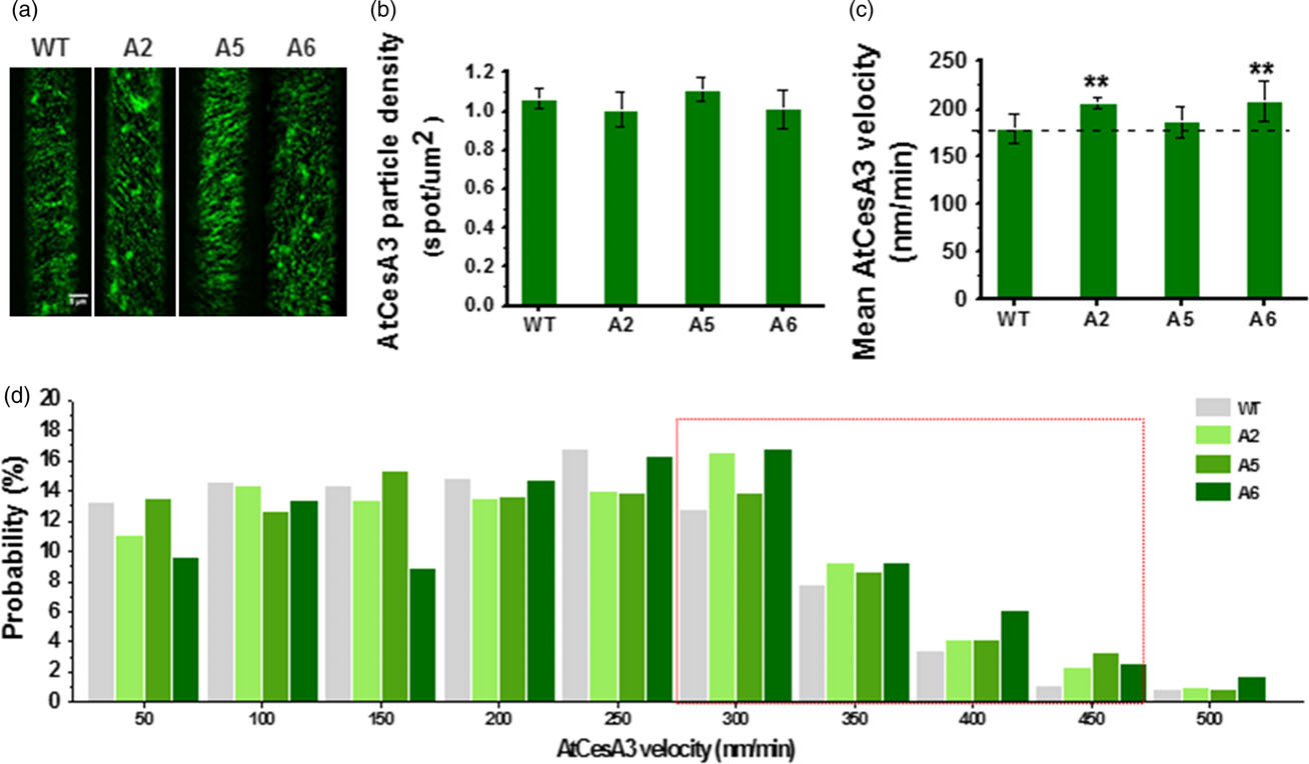
Increased dynamic movements of primary wall GFP‐CesA3 at plasma membrane in the *CesA6‐*like overexpressing transgenic seedlings. (a) Schematic images of GFP‐CesA3 dynamic movements in epidermal cells of D3 hypocotyls. Scale bar, 5 μm. (b–d) Density of GFP‐CesA3 particles measured as foci/μm^2^ (b), GFP‐CesA3 mean velocity measured as nm/min (c) and velocity distribution of GFP‐CesA3 particles (d). Data indicated means ± SD; 578–1257 CesA3 particles were detected with *n* ≥ 4 cells from four different seedlings for each genotypes; **P <* *0.01 by Student's *t*‐test.

To assess the behaviour of increased primary wall *CesAs* or the cellulose synthase complexes (CSCs), we crossed the three overexpressing lines (A2, A5 and A6) with the *proAtCesA3::GFP‐AtCesA3* marker line, in which primary wall CSC behaviour may be assessed (Desprez *et al*., 2007; Figure 3a). Compared to WT, we did not find any major differences in GFP‐CesA3 particle density at the plasma membrane between the overexpressing lines and control in D3 hypocotyls (Figure 3b). However, the GFP‐CesA3 particles in the plasma membrane moved significantly faster in the overexpressing lines, especially in A6 and A2 lines (Figure 3c). Notably, the three overexpressing lines showed a larger proportion of GFP‐CesA3 particles moving with speeds higher than 300 nm/min (Figure 3d). Thus, overexpression of the three *CesA6‐*like genes may cause an increase in CSC motility at the plasma membrane.

### Cellulose synthesis is enhanced in *Arabidopsis* transgenic seedlings

**Figure 4:**
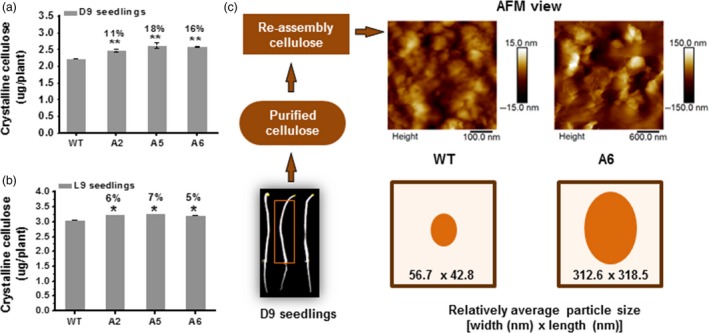
Enhanced cellulose synthesis in three *CesA6‐*like overexpressing seedlings. (a, b) Absolute crystalline cellulose contents in overexpressing seedlings; (a) D9 seedlings and (b) L9 seedlings. Bars indicated means ± SD (*n* = 3 biological replicates), and 100 seedlings were measured for each replicate; *P <* *0.05 and **P <* *0.01 by Student's *t*‐test; the differences in increased rates (%) were calculated by subtraction of values between overexpression transgenic lines and WT divided by WT. (c) Reassembly of macrofibrils from purified cellulose using atomic force microscopy (AFM). The relative average particle size (width × length) was calculated from randomly selecting ten particles in each image from three biological replicates.

CesA levels and movements typically correlate with cellulose production (Bringmann *et al*., 2012; Gutierrez *et al*., 2009; Paredez *et al*., 2006; Sanchez‐Rodriguez *et al*., 2017). To assess whether the transgenic lines produced more cellulose than WT, we measured crystalline cellulose levels in seedlings. Compared to WT, the three overexpressing lines (A2, A5 and A6) contained significantly increased levels of crystalline cellulose per plant in D9 and L9 seedlings (ranging from 5% to 11% increase; Figure 4a,b). In terms of cell wall compositions, D9 seedlings of the three overexpressing lines had higher crystalline cellulose and pectin levels and relatively lower hemicelluloses level per dry weight than those of WT (Figure S5a–c). Based on the monosaccharide composition analysis of total wall polysaccharides, all three transgenic lines showed significantly lower proportions of rhamnose, fucose and mannose, with variations of other monosaccharides, compared to WT (Figure S5d).

Furthermore, we examined properties of the cellulose by observing the reassembly of macrofibrils *in vitro* under atomic force microscopy (AFM) from D9 hypocotyls. The *CesA6* overexpressing lines exhibited apparent larger and egg‐shaped macrofibrils as compared to the WT material (five fold increase in size), suggesting that overexpression of *CesA6* genes can affect microfibril organization (Figure 4c). In summary, overexpression of the three *CesA6*‐like genes can enhance cellulose biosynthesis and influence the size of cellulose macrofibril aggregates *in vitro*, perhaps caused by the increased movement of primary wall CesAs.

### Cell elongation and division are enhanced in *Arabidopsis* transgenic seedlings

**Figure 5:**
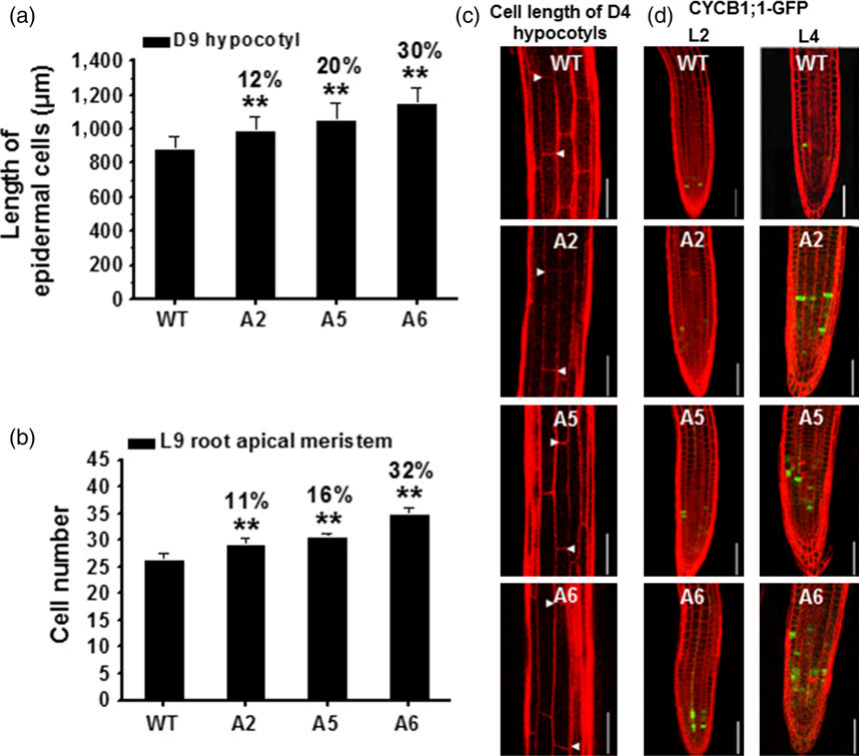
Enhanced cell elongation and division in three *CesA6‐*like overexpressing seedlings. (a, b) Measurement of basal longest epidermal cells of D9 hypocotyls (a) and cell number of L9 root apical meristem (b). Bars indicated means ± SD (*n* = 3 biological replicates), and at least 30 seedlings were measured for each replicate; **P <* *0.01 by Student's *t*‐tests. (c) Confocal laser scanning microscopy images of basal longest epidermal cells of D4 hypocotyls using propidium iodide (PI) staining (red‐fluorescent). Arrowheads indicated a single cell; scale bars, 100 μm. (d) Typical expression of G2/M‐specific marker *proAtCYCB1; 1::AtCYCB1; 1‐GFP* (green) of plant cell cycle in the root apical meristem using PI staining (red‐fluorescent). Scale bars, 75 μm.

To assess what aspects of seedling growth were enhanced by the *CesA6*‐like overexpression, we estimated cell elongation and division. We first measured the basal epidermal cells of D9 hypocotyls. These cells were significantly longer in the three *CesA6*‐like overexpression lines as compared to WT (Figure 5a,c). Because the number of epidermal cells in a single vertical cell file (parallel to the direction of growth) is genetically fixed to approximately 20 cells in *Arabidopsis* hypocotyls (Gendreau *et al*., 1997), we presumed that the increased hypocotyl lengths are mainly due to enhanced cell elongation in the transgenic plants. Root length is determined by both cell division and elongation (Dolan *et al*., 1993), and we therefore estimated the cortical cell numbers of root apical meristem from the quiescent centre (QC) to the transition zone (TZ) in L9 seedlings to assess the causes for the increased root growth (Beemster and Baskin, 1998). The transgenic lines contained more cells in this region as compared to WT, indicating an enhanced cell division in these lines (Figure 5b). To test this, we crossed the three *CesA6‐*like overexpressing lines with the *proAtCYCB1; 1::AtCYCB1; 1‐GFP* marker line, a classic G2 (interphase) to M (mitotic phase) specific marker of the cell division cycle (Ferreira *et al*., 1994; Ubeda‐Tomas *et al*., 2009). Analyses of the progeny of these crosses revealed that the transgenic lines had more cells undergoing division than WT in L4 root tips (more green fluorescent foci visible in the roots; Figure 5d). Thus, overexpression of the three *CesA6*‐like genes can enhance both cell elongation and division in *Arabidopsis* seedlings.

To investigate how overexpression of the *CesA6*‐like *CesAs* influenced general gene expression, we performed RNA sequencing experiments of 6‐day‐old dark‐grown (D6) WT, A2, A5 and A6 transgenic seedlings. Many genes included under Gene Ontology and Biological Process terms (GO and BP terms) associated with cell growth and cellulose synthesis showed clear differences in their expression in the transgenic lines, compared to WT (Figure S6 and Table S3). These changes might indicate that the increase in the primary wall *CesA* genes could affect plant growth in other ways than simply making more cellulose. This is compatible with the mutations in *THESEUS* that increase the growth of the *prc1‐1* mutant (affecting *CesA6*) without changing the amounts of cellulose (Hematy *et al*., 2007).

### Biomass yields are increased in three CesA6‐like transgenic mature plants

**Figure 6:**
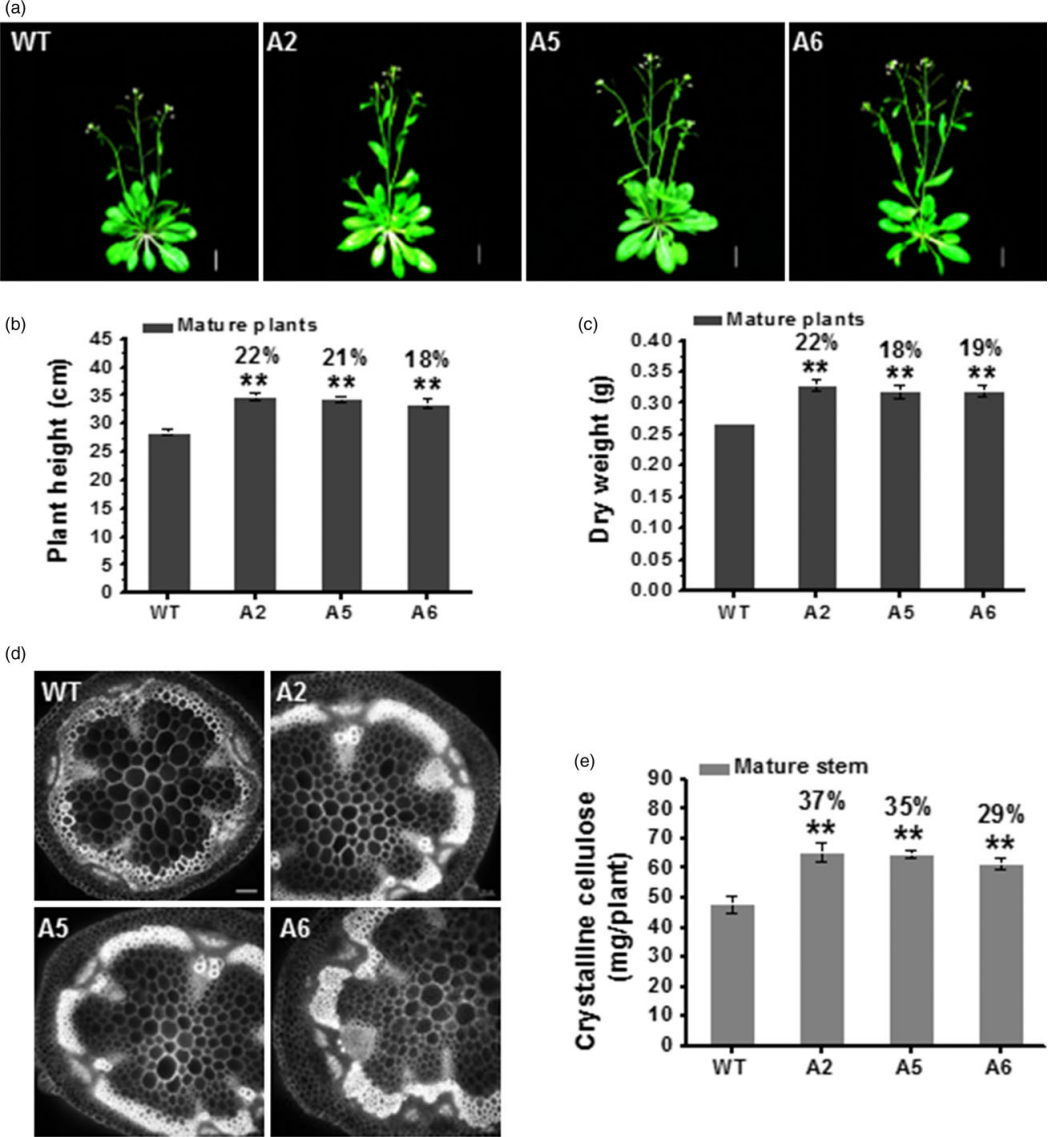
Increased cellulose synthesis and biomass production in three *CesA6‐*like overexpressing plants. (a) Plant phenotypes at the flowering stage of indicated plant genotypes. Scale bars, 15 mm. (b, c) Plant height (b) and dry weight (c) of 7‐week‐old mature plants. Bars indicated means ± SD (*n* = 3 biological replicates), and at least 30 plants were measured for each replicate; **P <* *0.01 by Student's *t*‐test. (d) Transverse sections of 1st internode stem at the bolting stage under epifluorescence microscopy using calcofluor staining. Scale bars, 50 μm. (e) The absolute crystalline cellulose contents per plant in 7‐week‐old inflorescence stems of mature plants. Bars indicated means ± SD (*n* = 3 biological replicates); **P <* *0.01 by Student's *t*‐test.

While the *CesA6*‐like transgenic plants were grown on soil, we found that these plants were taller and had more dry weight as compared to WT after 7 weeks (Figure 6a–c). We undertook microscopic observations of transverse sections of 1^st^ internode stem and stained with calcofluor, which stains glucans including cellulose (Haigler *et al*., 1980). We observed relative stronger calcofluor fluorescence in the *CesA6*‐like overexpressing lines than that of WT (Figure 6d). We then analysed cell wall composition of the 7‐week‐old inflorescence stems of mature plants. We found that the three *CesA6*‐like genes overexpressing lines contained more crystalline cellulose per plant (ranging from 29% to 37% increase) as compared to WT (Figure 6e). These overexpressing transgenic plants also showed significantly higher crystalline cellulose and lignin levels with relatively lower hemicelluloses content per dry weight, compared to WT (Figure S7a,c,d), whereas no significant difference was found in relative pectin levels among all transgenic lines and WT (Figure S7b). Moreover, based on monosaccharide composition analysis of total wall polysaccharides, all mature transgenic plants showed a significant increase in xylose and decrease in other monosaccharides, as compared to WT (Figure S7e). These data thus indicate that the enhanced biomass yields are mainly due to an increase in cellulose and lignin levels in the three *CesA6*‐like genes overexpressing lines.

### Secondary wall thickness is increased in transgenic mature plants

**Figure 7:**
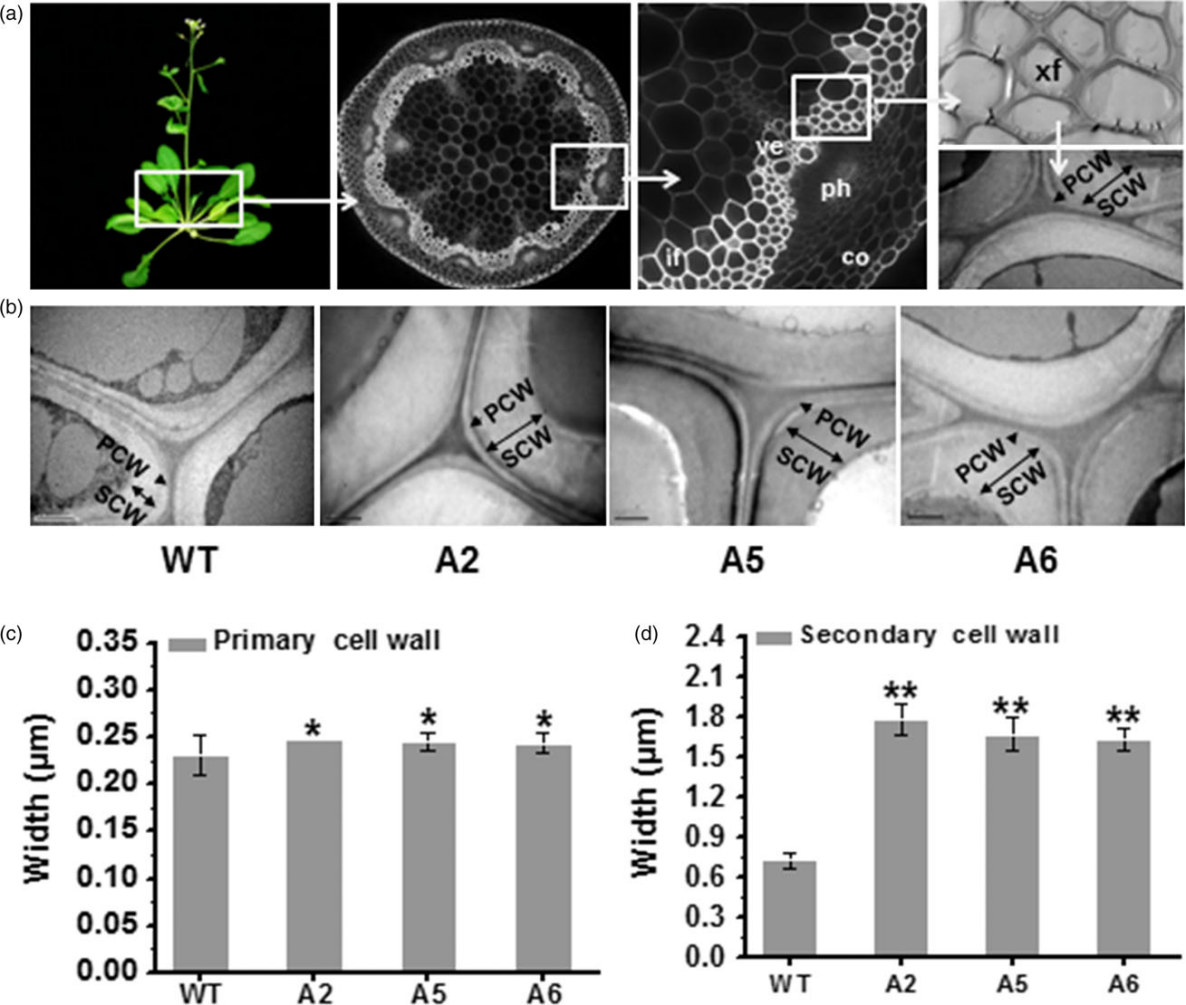
Enhanced secondary cell wall deposition in three *CesA6‐*like overexpressing plants. (a) Schematic diagram for observing the sclerenchyma cell walls in the 1st internode stem of 7‐week‐old *Arabidopsis* plants using transmission electron microscopy (TEM). PCW, primary cell wall; SCW, secondary cell wall; co, cortex; ph, phloem; ve, vessel; xf, xylary fibre; if, interfascicular fibre. (b) Cell wall observations in xf tissues. Scale bars, 1 μm. (c, d) PCW and SCW widths. Bars indicated means ± SD (*n* = 3 biological replicates), and at least 60 cell walls were measured for each replicate; *P <* *0.05 and **P <* *0.01 by Student's *t*‐test.

Because plant secondary cell walls represent the major biomass production site (Fan *et al*., 2017; Li *et al*., 2017), we applied transmission electron microscopy (TEM) to observe the xylary fibre (xf) cells (the sclerenchyma cells) in the 1^st^ internode stem of 7‐week‐old *Arabidopsis* plants (Figure 7a). The TEM images revealed that the overexpressing lines had much thicker cell walls of xf tissues (Figure 7b), which is largely comprised of secondary walls. Quantification of the cell wall width showed that the overexpressing lines exhibited thicker primary walls than those of WT (Figure 7c), consistent with their increased cellulose levels and large macrofibrils in reassembly assays of seedlings (Figure 4). Notably, they also had remarkably increased secondary cell wall widths (more than two fold) compared with WT (Figure 7d). Hence, overexpression of the *AtCesA6*‐like genes can increase both primary and secondary wall deposition.

We also examined the impact of *CesA3* and *CesA9* on secondary cell wall formation in transgenic plants (A3 and A9; Figure S8a). Unlike the *CesA2* and *CesA5*, overexpression of *CesA3* or *CesA9* did not increase secondary cell wall widths, consistent with its inability to enhance young seedling growth (Figure S3a,b) and plant growth (Figure S8b). Surprisingly, despite the similar phenotypes observed in the young seedlings in comparison with WT (Figure S3a), the *CesA7* overexpressing plants (A7) exhibited incomplete xf cell walls (Figure S8a), suggesting that simply overproducing secondary *CesA* genes are unlikely to increase secondary wall deposition. The incomplete walls may be due to defects in cell wall integrity or may be cosuppression of other secondary wall genes (Figure S8b).

### Wall mechanical strength is increased in transgenic mature plants

**Figure 8:**
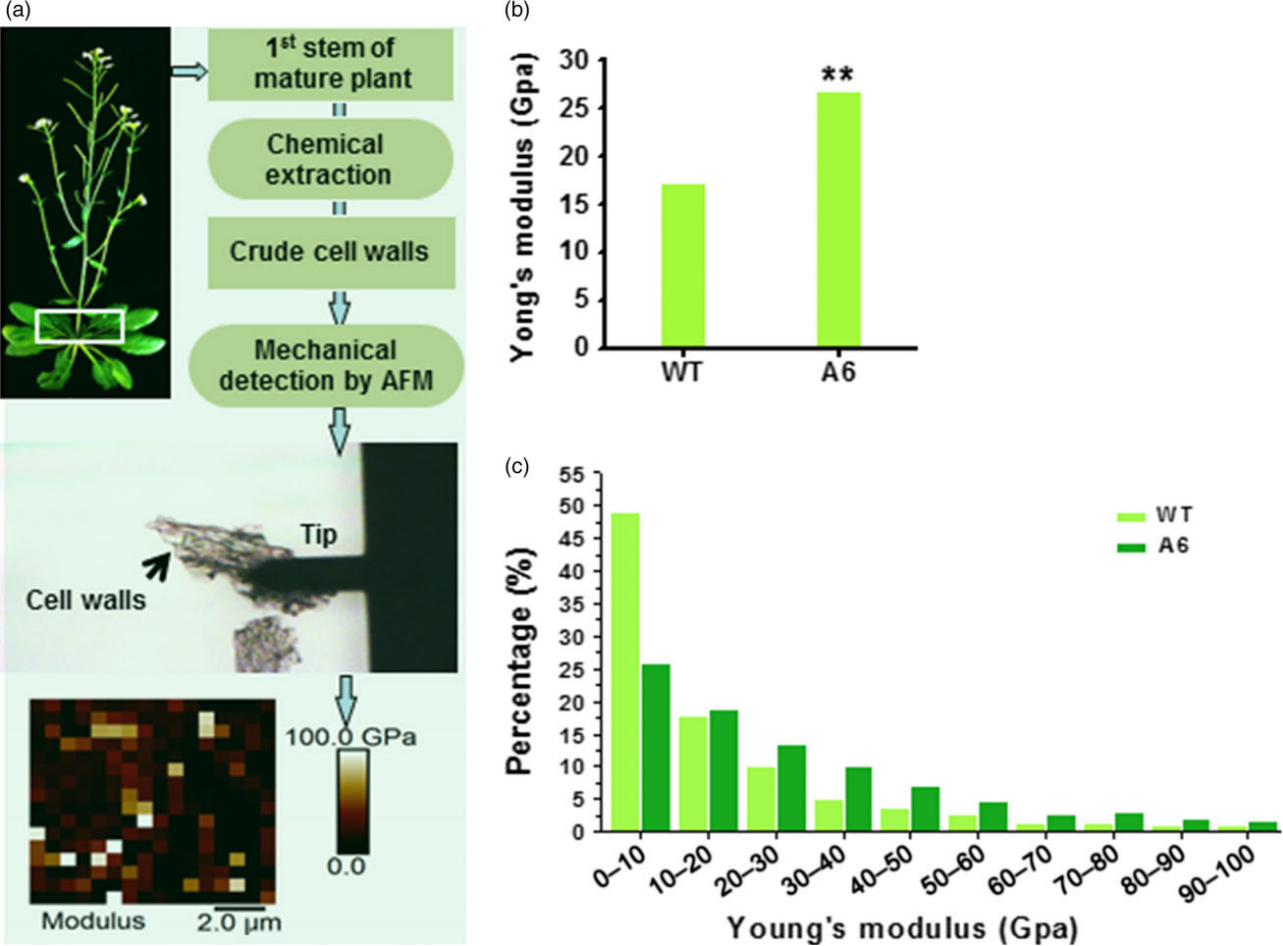
Increased mechanical strength of reassembled crude cell walls in 1st internode stems of the *CesA6‐*like overexpressing plants. (a) Schematic flow for mechanical force measurements (Young's modulus) of reassembled crude cell walls in 1st internode stems of 7‐week‐old plants using AFM. (b, c) Mean value (b) and distribution (c) of Young's modulus of crude cell walls. Bars indicated means of two biological replicates; 30 cell segments (*n* = 30) were measured for each replicate; *P <* *0.05 and **P <* *0.01 by Wilcoxon test.

As cell walls provide plants with mechanical strength (Fan *et al*., 2017), we extracted crude cell walls from the 1^st^ internode stem of 7‐week‐old plants and detected their wall forces (Young's modulus) using AFM technology (Figure 8a). Compared to WT, the *CesA6* overexpressing plants exhibited significantly enhanced mean mechanical strength (Figure 8b) with a higher proportion of Young's modulus values in the range from 10 to 100 GPa (Figure 8c). Therefore, the *CesA6* overexpression plants had relatively higher mechanical strength in the basal stems of plants, most likely due to the enhanced secondary wall synthesis.

## Discussion

Plant cell walls determine the size and shape of many different cell types that form the tissues and organs of a plant (Farrokhi *et al*., 2006; Keegstra, 2010; Zhang *et al*., 2009). Cellulose is a prominent and load‐bearing component of most plant cell walls and is of great relevance for many industries (Somerville, 2006). Thus, changes in the cellulose synthesis producing capacity of plants would be very tractable and may provide suitable traits for biomass‐producing crop plants. Several studies have attempted to increase cellulose synthesis by overexpressing different secondary wall *CesAs* with limited success. For instance, attempts were made in barley and poplar, which largely resulted in the silencing of both transgenes and the endogenous genes (Joshi *et al*., 2011; Tan *et al*., 2015). In this study, we reported that increasing the expression levels of certain primary wall *CesA6*‐like *CesAs*, that is *CesA2*,* CesA5* and *CesA6*, can improve cellulose synthesis and plant growth in *Arabidopsis*. However, overexpression of *CesA3*,* CesA9* and *CesA7* did not lead to improved plant growth in our hands, although the expression of the transgenes and the corresponding endogenous genes appeared to be maintained by Q‐PCR analyses (Figure S3c). However, despite of a technique difficulty, it remains interesting to examine those three CesA proteins in the transgenic plants and their potential impacts on CSCs formation. In addition, overexpression of *CesA7* could affect cell wall integrity and lead to a relatively decreased biomass yield in the mature transgenic plants (Figure S8), which perhaps supports other unsuccessful attempts to increase biomass yields by overexpressing secondary wall *CesAs* (Joshi *et al*., 2011; Tan *et al*., 2015). The *CesA7* overexpressing plants might be interesting experimental samples for exploring how secondary wall deposition or defects affect cell wall integrity and plant growth in the future.

Mutations in primary wall *CesAs* typically lead to reduced cell elongation and loss of anisotropic growth (Bischoff *et al*., 2011; Cano‐Delgado *et al*., 2003; Chen *et al*., 2010, 2016; Fagard *et al*., 2000; Fujita *et al*., 2013). Here, we demonstrated that overexpressing *CesA2*,* CesA5* or *CesA6* genes led to enhanced cell elongation, possibly due to increased cellulose synthesis. Apart from the positive influence on cell elongation, we also observed an increased number of cells undergoing division in transgenic seedling roots (Figure 5). It is well established that primary wall cellulose‐deficient plants have incomplete cell walls, for example *prc1‐1* affecting *AtCesA6*, which most likely indicates a failure in completing the cell plate during cell division (Fagard *et al*., 2000). Indeed, primary wall CesAs are trafficked to and from the growing cell plate during its progression, corroborating a function of the primary wall CesAs in this process (Miart *et al*., 2014). In addition, cellulose synthase‐like (CSL) gene family members promote cell division in maize leaves, rice and *Arabidopsis* roots (Gu *et al*., 2016; Hunter *et al*., 2012; Yoshikawa *et al*., 2013). Nevertheless, how the *CesA* expression levels regulate the rate of cell division will be interesting to unravel in future studies.

The RNA sequencing data of the three *CesA* overexpression lines revealed many differentially expressed genes (DEGs) that are associated with cell growth [e.g. *EXPA11* (expansin), *CSI3* and *CSI1/POM2* (microtubule and cellulose synthase‐associated proteins), *PIF1* and *PIF7* (phytochrome‐interacting factor, light‐mediated suppression of hypocotyl elongation), *CYCP1; 1* and *CYCP2; 1* (cyclin‐related proteins), *SMR5* (DNA replication), and others] (Table S3), as compared with WT. Hence, these results indicate that other biological processes, besides cellulose synthesis, may also influence the growth of the transgenic plants.

The primary wall CesAs (at least CesA1, CesA3 and CesA6) can interact both *in vitro* and *in planta* with all secondary wall CesAs (CesA4, CesA7 and CesA8; Carroll *et al*., 2012), and the authors speculated that sufficient parallels exist between the primary and secondary complexes for cross‐functionality. Hence, there is a possibility that mixed complexes of primary and secondary wall CesAs could occur at certain times during plant growth (Carroll *et al*., 2012). In addition, CesA2 and CesA5 play major roles in secondary wall cellulose production in seed mucilage (Griffiths *et al*., 2014; Mendu *et al*., 2011; Sullivan *et al*., 2011). While it still is difficult to conclude how secondary wall synthesis is improved in the *CesA6*‐like overexpressing plants, it is possible that the enhanced primary wall CesAs could promote or interact with secondary wall CesAs, and participate in secondary cell wall synthesis, or perhaps that the increased cell elongation and division provide larger cell space for cell wall deposition.

**Figure 9:**
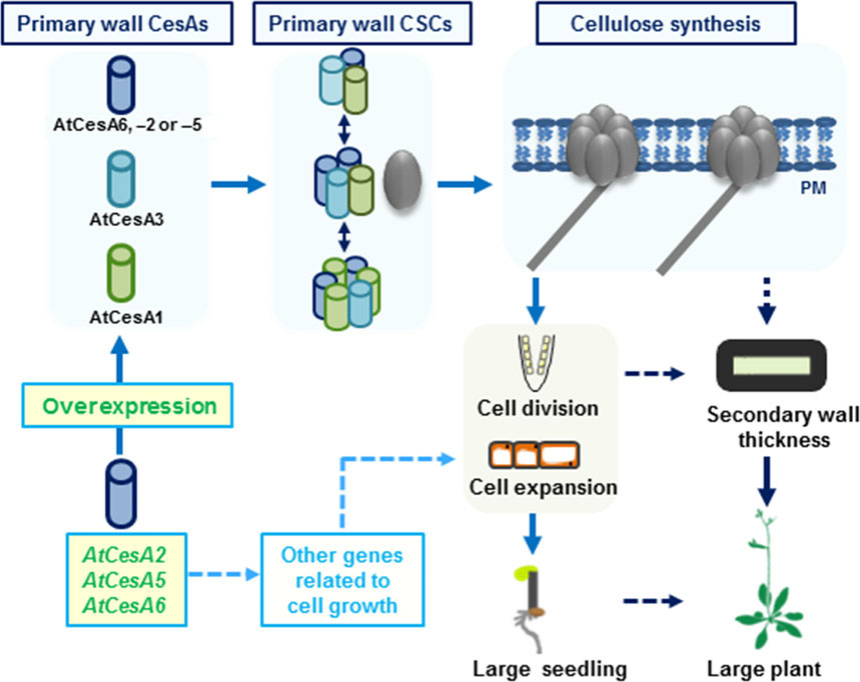
A hypothetical model of how overexpression of three *CesA6*‐like genes affect plant growth for biomass production. The model highlights that overexpressing any of the *CesA2*,* CesA5* or *CesA6* genes can regulate the expression of other primary wall *CesA* genes and enhance the CSCs movement to produce more cellulose. It also outlines that the overexpression of three *CesA6*‐like genes can influence the expression of many other genes associated with cell growth, leading to enhanced cell expansion and division in *Arabidopsis* seedlings. It finally concludes that the increased cell growth could further enhance secondary cell wall deposition that influences the mechanical strength and biomass production.

In conclusion, we propose a hypothetical model for how the three *CesA6‐*like genes affect cellulose biosynthesis, cell wall deposition and plant cell growth for biomass production (Figure 9): (i) Overexpressing any of the *CesA2*,* CesA5* or *CesA6* genes enhances the expression of other major primary wall *CesA*s (as well as several cellulose‐related genes like *KORRIGAN2*,* SUS1*,* COBRA*,* CSI1* and *CSI3*) to produce more cellulose. (ii) The three *CesA6*‐like genes overexpressions cause changes in expression of genes related to cell growth that may influence cell expansion and division in *Arabidopsis* seedlings. (iii) The enhanced cellulose synthesis and cell growth lead to an increase in secondary cell wall deposition, either directly or indirectly, and to improved mechanical strength and plant growth.

## Experimental procedures

### Plant materials and growth conditions

Complete *AtCesA* (*AtCesA6*,* AtCesA2*,* AtCesA5*,* AtCesA3*,* AtCesA9* and *AtCesA7*) coding regions (cDNAs) were cloned and driven by double *35S* promoter in the binary vector pD1301s plasmid (Table S2). The lines transformed with an EV were used as control. Transgenic plants were generated by introducing the constructs into an *Agrobacterium tumefaciens* strain GV3101, and transformation was carried out by floral dipping method (Zhang *et al*., 2006). T1 transgenic seedlings were selected on 1/2 MS medium containing 50 mg/L hygromycin, and further confirmed by RT‐PCR or Q‐PCR. More than three hygromycin‐resistant lines (independent transformation events) for each construct were selected to and propagated to homozygous states. Phenotypic characterization was performed on T5 homozygous transgenic lines.

*Arabidopsis* seeds were surface sterilized using 75% ethanol for 4 min, 10% sodium hypochlorite with 0.01% Triton X‐100 for 3 min and washed in sterile water several times, then imbibed at 4 °C in the dark in sterile water containing 0.1% agar for 3 days and germinated on plates containing half MS media (1% sucrose; pH 5.8) in 1% agar. Plates were incubated in a near‐vertical position at 22 °C under light‐grown condition (16‐h light/8‐h dark) for photomorphogenesis or dark‐grown condition (24‐h dark) for skotomorphogenesis. The seedlings were transplanted to the soil after the second real leaf is clearly visible.

### RNA extraction and Q‐PCR measurement

Seedlings were germinated and grown on 1/2 MS medium for indicated number of days under light‐ or dark‐grown conditions, and seedlings (hypocotyls, roots) were harvested in liquid nitrogen. Total RNA was isolated from the collected tissues using Trizol reagent (Invitrogen, Carlsbad, CA). First‐strand cDNA was obtained using OligodT and M‐MLV reverse transcriptase (Promega, Madison, WI, USA). Q‐PCR amplification was carried out on a Bio‐Rad MyCycler thermal cycler with SYBER Premix ExTaq (TakaRa, Tokyo, Japan) according to the manufacturer's instruction, and *AtGAPDH* was used as the internal control. The PCR thermal cycle conditions were as follows: one cycle of 95 °C for 2 min, followed by 45 cycles of 95 °C for 15 s, 58 °C for 15 s and 72 °C for 25 s. The expression value of *GAPDH* was defined as 100, and the expression level of *CesA* genes was thus normalized to the expression level of *GAPDH*. All of the primers used in these assays are listed in Table S1. Three biological replications were performed.

### Total proteins extraction and Western blot analysis

D9 seedlings were ground to a fine powder in liquid nitrogen, and 1.0 g powder was extracted using Plant Total Protein Lysis Buffer (Sangon Biotech, Shanghai, China; PL011) (1 mL Solution A, 10 μL Solution B, 10 μL Solution C) with protease inhibitors (1.0 mm Phenylmethanesulfonyl fluoride, 1.0 μm pepstatin A and 1.0 μm leupeptin). The extracts were transferred to 2‐mL tubes under ultrasonic treatment on the ice for 20 min. The suspension liquid was incubated for 30 min at 4 °C under continuous stirring in the presence of 50 μL 1% digitonin or 100 μL 20% Triton X‐100. The homogenate was centrifuged at 12 000 *g* for 30 min at 4 °C. The protein concentration in the supernatant was determined using bicinchoninic acid Protein Assay Kit (Qcbio S&T). The AtCesA2, AtCesA5 and AtCesA6 protein levels were detected by Western blot analysis as described previously (Li *et al*., 2017). Purification of primary antibodies was performed using Protein A‐Agarose and detected by Western blot analysis in WT (Figure S2a). Antibody dilutions were performed as 1 : 250, 1 : 125 and 1 : 30 for AtCesA6, AtCesA2 and AtCesA5 antibodies, respectively.

### Velocity and density measurements of GFP‐CesA3 at the plasma membrane

To investigate the CesA dynamics in seedlings, the *Arabidopsis* transgenic overexpression lines were crossed with CesA marker *proAtCesA3::GFP‐AtCesA3* (Desprez *et al*., 2007). F1 seeds were surface sterilized and sown on 1/2 MS (plus 1% sucrose) plates where the seeds were stratified at 4 °C for 2 days. Afterwards, the plates were transferred to the growth chamber (22 °C, light/dark 16 h/8 h), exposed to light about 3 h and then covered with the aluminium foil. Etiolated hypocotyls were used for imaging after vertically grown for 3 days, and images were obtained from epidermal cells within 2 mm below the apical hook. The enhanced etiolated hypocotyl phenotype of the overexpression lines was confirmed to assure that gene silencing of the overexpression constructs was not occurring.

The CesA velocity measurement and analysis were performed as described previously (Ivakov *et al*., 2017). The density of CesAs was measured with the plugin TrackMate (http://fiji.sc/TrackMate) in Fiji (http://fiji.sc/Fiji). The time lapses were used for analysis. Median filter was applied. The particle diameter and the maximum/minimum signal intensity were used to avoid the interference of the noise and Golgi‐localized CesAs. Significant differences were performed by Student's *t*‐test. 578 – 1257 CesA3 particles were detected with *n* ≥ 4 cells from four different seedlings for each genotype.

### Observation of cellulose macrofibrils by atomic force microscopy (AFM)

The purified cellulose sample fractionations of D9 hypocotyls were performed as described previously (Li *et al*., 2017), with some minor modifications that hypocotyls were milled into fine powder under liquid nitrogen and then add 8% NaClO_2_ (10 mL). The precipitated residue was treated with 4 M KOH for 1 h, washed with distilled water six times and resuspended in water for AFM scanning. The cellulose samples were suspended in ultrahigh‐purity water and placed on mica using a pipette. The mica was glued onto a metal disc (15 mm diameter) after removal of extra water under nitrogen and then placed on the piezo scanner of AFM (MultiMode VIII; Bruker, Santa Barbara, CA). AFM imaging was carried out in ScanAsyst‐Air mode using BrukerScanAsyst‐Air probes (tip radius, 2 nm and silicon nitride cantilever; spring constant, 0.4 N/m) with a slow scan rate of 1 Hz. All AFM images were 3rd‐flattened and analysed quantitatively using NanoScope Analysis software (Bruker). Three biological replications were performed each experiment, and 10 dots of each AFM image were randomly selected to measure the width (nm) × length (nm) by NanoScope Analysis software (Bruker). The average particle length/width of each image was calculated from the selected ten particles (*n* = 10).

### Hypocotyl, root and cell length measurements

To observe hypocotyl and root growth, *Arabidopsis* seedlings were scanned using an HP Scanjet 8300 scanner at 600 dpi, the hypocotyl length of vertically grown seedlings was measured from hypocotyl base to the apical hook and the root length was measured (root tip to hypocotyl base) using ImageJ 1.32j (http://rsb.info.nih.gov/ij/). Two‐tailed *t*‐tests were performed with Microsoft Excel software. For images of epidermal cell patterns, D9 hypocotyls were mounted and images of epidermal cells were viewed using differential interference contrast (80i; Nikon, Japan). At least three biological replicates were performed each experiment, and more than 30 seedlings were measured each genotype. Cell lengths in recorded images were quantified using ImageJ, and epidermal cells of hypocotyl were visualized under confocal laser scanning microscopy (p58; Leica, Leica Microsystems, Nussloch, Germany) using 4‐day‐old dark‐grown (D4) hypocotyls incubated in the dark for 10 min in a fresh solution of 15 mm (10 mg/mL) propidium iodide (PI; Naseer *et al*., 2012). PI was excited at 488 nm, and fluorescence was detected at 600–700 nm.

### Cell division observation

Root meristem size was highlighted as the distance between the quiescent centre (QC) and the transition zone (TZ, indicating the position of the first elongating cortical cell), and the number of cortical cells was counted in a file extending from QC to TZ (Beemster and Baskin, 1998). To count the number of cortical cells, L9 root tips were mounted, and images viewed by differential interference contrast (80i; Nikon, Japan). At least three biological replications were performed each experiment, and more than 30 seedlings were measured each genotype. To visualize cell cycle progression in living cells, G2/M‐specific marker *proAtCYCB1;1::AtCYCB1;1‐GFP* (Ubeda‐Tomas *et al*., 2009) was crossed with different homozygous *AtCesA6*‐like transgenic lines. Measurements of F1 hybrid seedlings were performed using confocal images of light‐grown roots stained with PI. GFP was excited at 473 nm, and fluorescence was detected at 485–545 nm.

### Observation of cell wall structures by transmission electron microscopy (TEM)

TEM was used to observe cell wall structures in the xylary fibre (xf) cells of the 1st inflorescence stems of 7‐week‐old plants. The samples were post‐fixed in 2% (w/v) osmium tetroxide (OsO_4_) for 1 h after extensively washing in the PBS buffer and embedded with Super Kit (Sigma‐Aldrich, St. Louis, MO, USA). Sample sections were cut with an Ultracut E ultramicrotome (Leica) and picked up on formvar‐coated copper grids. After poststaining with uranyl acetate and lead citrate, the specimens were viewed under a Hitachi H7500 transmission electron microscope. The width of three relatively fixed points on each cell wall was measured using ImageJ. More than 60 cell walls for each *t* genotype were measured. Significance differences were performed by Student's *t*‐test. Three biological replications were performed.

### Crude cell wall extraction and mechanical force measurement by AFM

The basal (1 cm) inflorescence stems from 7‐week‐old plants were ground under liquid nitrogen and then incubated at 70 °C in 96% (v/v) ethanol for 30 min. The pellet was successively washed with absolute ethanol, twice with 2 : 3 (v/v) chloroform: methanol, then once each with 65% (v/v), 80% (v/v) and absolute ethanol, and the remaining pellet was freeze‐dried as crude cell wall material. The crude cell wall material was suspended in ultrahigh‐purity water, placed on new mica using a pipette and dried in air overnight. The mica was glued onto a metal disc (15 mm diameter) and placed on the piezo scanner of an AFM (MultiMode VIII; Bruker). A hard tip (RTESP; Bruker) with radius of 8 nm, and spring constant of 40 N/m was used in the mechanical properties measurement. The precise spring constant was corrected by Sader method, and the deflection sensitivity was average determined by measuring a set of force–distance curves on the mica. The scan size was 10 μm × 10 μm, and 16 × 16 FD curves were collected for every measurement, and 10 different cell segments were randomly selected for mechanical measurements each sample. The Young's modulus was calculated using Hertz model of the NanoScope analysis software, and Wilcoxon test was used to test significance of average Young's modulus (He *et al*., 2015). Two biological replications were performed each experiment.

### Plant height and dry weight measurement

The homozygous lines were transplanted into soil as individual plant per basin; the plants were grown in a glasshouse at 22 °C under light‐grown condition for 7 weeks in a fully randomized experimental design. The plant height was measured from the basal stem to the peaks of the mature *Arabidopsis* plants. Harvested 7‐week‐old inflorescence stems per plant, then dried under suitable temperature (55 °C) for 3–5 days and finally weighed by analytical balance. Three biological replications were performed each experiment, and more than 30 plants were measured each genotype. Significance analysis was performed by Student's *t*‐test.

### Plant cell wall composition fractionation and determination

Plant cell wall fractionations and determination were performed as described previously (Jin *et al*., 2016), with some minor modifications for crystalline cellulose extraction. For crystalline cellulose extraction, one hundred D9 or L9 seedlings or the dry biomass powder of 7‐week‐old inflorescence stems (40 mesh) samples (0.1–1.0 g) were suspended in 5.0 mL acetic acid–nitric acid–water (8 : 1 : 2, v/v/v) and heated for 1 h in a boiling water bath with stirring every 10 min. After centrifugation, the pellet was washed several time with 5.0 mL water and the remaining pellet was defined as crystalline cellulose sample. Total lignin was determined as described previously (Sun *et al*., 2017). At least three biological replications were performed.

### Determination of monosaccharide composition of total wall polysaccharides by GC‐MS

Both the seedlings and the dry biomass powder (40 mesh) samples (0.1–1.0 g) were washed twice with 5.0 mL buffer and twice with 5.0 mL distilled water. The remaining pellet was stirred with 5.0 mL chloroform–methanol (1 : 1, v/v) for 1 h at 40 °C and washed twice with 5.0 mL methanol, followed by 5.0 mL acetone. The pellet was washed once with 5.0 mL distilled water. The remaining pellet was added with 5.0 mL aliquot of DMSO–water (9 : 1, v/v), vortexed for 3 min and then rocked gently on a shaker overnight. After centrifugation, the pellet was washed twice with 5.0 mL DMSO–water and then with 5.0 mL distilled water three times. The remaining pellet was defined as total wall polysaccharides and confirmed by determining monosaccharide composition with GC‐MS as described previously (Xu *et al*., 2012). Three biological replications were performed.

### RNA sequencing and analysis

Total RNA was isolated from the 6‐day‐old dark‐grown (D6) seedlings (four samples, two biological replications) using Trizol reagent (Invitrogen). The RNA was checked for purity before performing RNA sequencing using NanoDrop 2000 (NanoDrop, Thermo‐Fisher, USA). RNA samples with RNA integrity numbers >8 were selected for library preparation, and RNA concentration was detected on Qubit 2.0 (Invitrogen). The optimized total RNA of 0.1–4 μg was used for this protocol. For cDNA library construction, total RNA was processed using a TruSeq™ RNA Sample Preparation Kit (Illumina, Tokyo, Japan) according to the manufacturer's instructions. All samples were sequenced using an Illumina HiSeq 2000 sequencer (Anders *et al*., 2015).

Raw reads were described previously (Mortazavi *et al*., 2008). DEGs were identified by applying the statistical tests for the group between transgenetic lines with wide type and performed by the DESeq R package (1.12.0). Then, *P* values were adjusted using the Benjamini and Hochberg method. A corrected *P* value of 0.001 and log2 (fold change) of 1 was set as the thresholds for significant differential expression (Anders and Huber, 2010).

Gene ontology (GO) terms of differential expressed genes were derived from agriGO. GO subontology ‘biological process’ (GO‐BP) was used for the gene‐set enrichment analysis. TopGO from Bioconductor in R (http://www.r-project.org/) was used to identify enriched GO terms (Du *et al*., 2010). This was performed for all DEGs. The enrichment analysis of GO‐BP terms relative to its expectation was performed using a weighted method in combination with Fisher's exact test.

### Microscopy observation

Seven‐week‐old *Arabidopsis* 1st inflorescence stems were embedded with the 4% agar and then cut into sections of 100 μm thick by microtome (VT1000S; Leica). Stem sections were stained in calcofluor for 3 min and then rinsed, mounted in water, and observed and photographed under epifluorescence microscopy (Olympus BX‐61, *Retiga*‐4000DC digital camera).

## Competing financial interests

The authors declare no competing financial interests.

## Supporting information


**Figure S1** *AtCesAs* expression patterns in hypocotyls and roots of *Arabidopsis* seedlings.
**Figure S2** Analyses of AtCesA2, ‐5 and ‐6 protein levels in *Arabidopsis* seedlings.
**Figure S3** Observations of seedlings in other *CesA* genes overexpression lines.
**Figure S4** Q‐PCR analyses of *CesA* genes in L9 roots of three *CesA6‐*like genes overexpressing lines.
**Figure S5** Cell wall compositions of D9 seedlings.
**Figure S6** Altered expression of genes associated with cell growth by RNA sequencing.
**Figure S7** Cell wall compositions of 7‐week‐old inflorescence stems of mature plants.
**Figure S8** Observations of plants in other *CesA* overexpression lines.
**Table S1** Q‐PCR primers.
**Table S2** Primers for overexpression vector construction.

**Table S3** Differentially expressed genes (DEGs) in D6 overexpressing seedlings by RNA sequencing.
