# Supplementary material for: Three AtCesA6‐like members enhance biomass production by distinctively promoting cell growth in Arabidopsis
Source: Plant Biotechnol J. 2017 Oct 23;16(5):976–88. doi: 10.1111/pbi.12842 (PMC5902768; doi:10.1111/pbi.12842)
Supplement: Supplementary file 1 — Figure S1 AtCesAs expression patterns in hypocotyls and roots of Arabidopsis seedlings. Figure S2 Analyses of AtCesA2, ‐5 and ‐6 protein levels in Arabidopsis seedlings. Figure S3 Observations of seedlings in other CesA genes overexpression lines. Figure S4 Q‐PCR analyses of CesA genes in L9 roots of three CesA6‐like genes overexpressing lines. Figure S5 Cell wall compositions of D9 seedlings. Figure S6 Altered expression of genes associated with cell growth by RNA sequencing. Figure S7 Cell wall compositions of 7‐week‐old inflorescence stems of mature plants. Figure S8 Observations of plants in other CesA overexpression lines. Table S1 Q‐PCR primers. Table S2 Primers for overexpression vector construction. [file PBI-16-976-s002.docx]

*Supplementary Information*

**Supplementary figures**

**
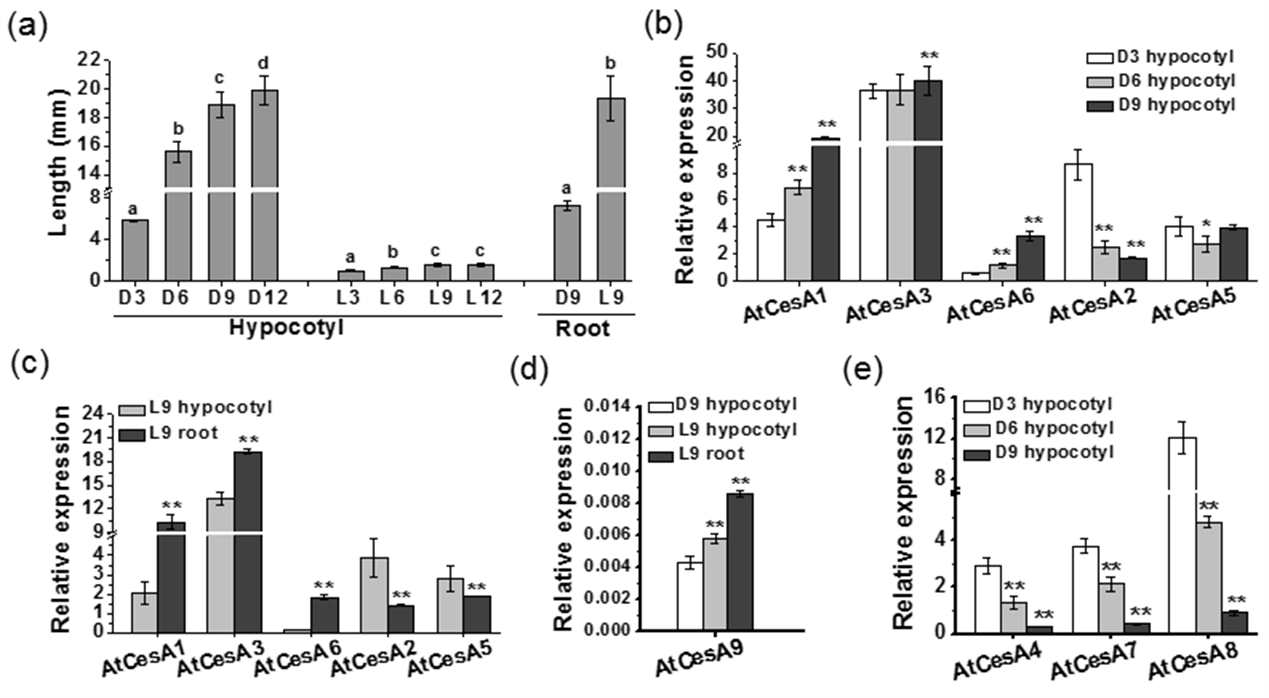
**

**Figure S1.** *AtCesAs* expression patterns in hypocotyls and roots of *Arabidopsis* seedlings. (a) Hypocotyl and root lengths of *Arabidopsis* wild type (WT; Col-0) seedlings grown in dark (D) or light (L) from 3 days (D3) to 12 days (D12) after sowing. Bars indicated means ± SD (n=3 biological replicates), and at least 30 plants were measured for each replicate; LSD (Least Significant Difference) test is used for multiple comparisons. Different letters above bars indicate that the means differ according to analysis of variance and LSD test (*P* < 0.01). (b-e) Q-PCR analyses of *CesA1-CesA9* endogenous gene expression levels using total RNA extracted from samples in (a); *GAPDH* used as the internal control and the expression value of *GAPDH* defined as 100. Bars indicated means ± SD (n=3 biological replicates); *** P* < 0.01 by Student’s t-test.


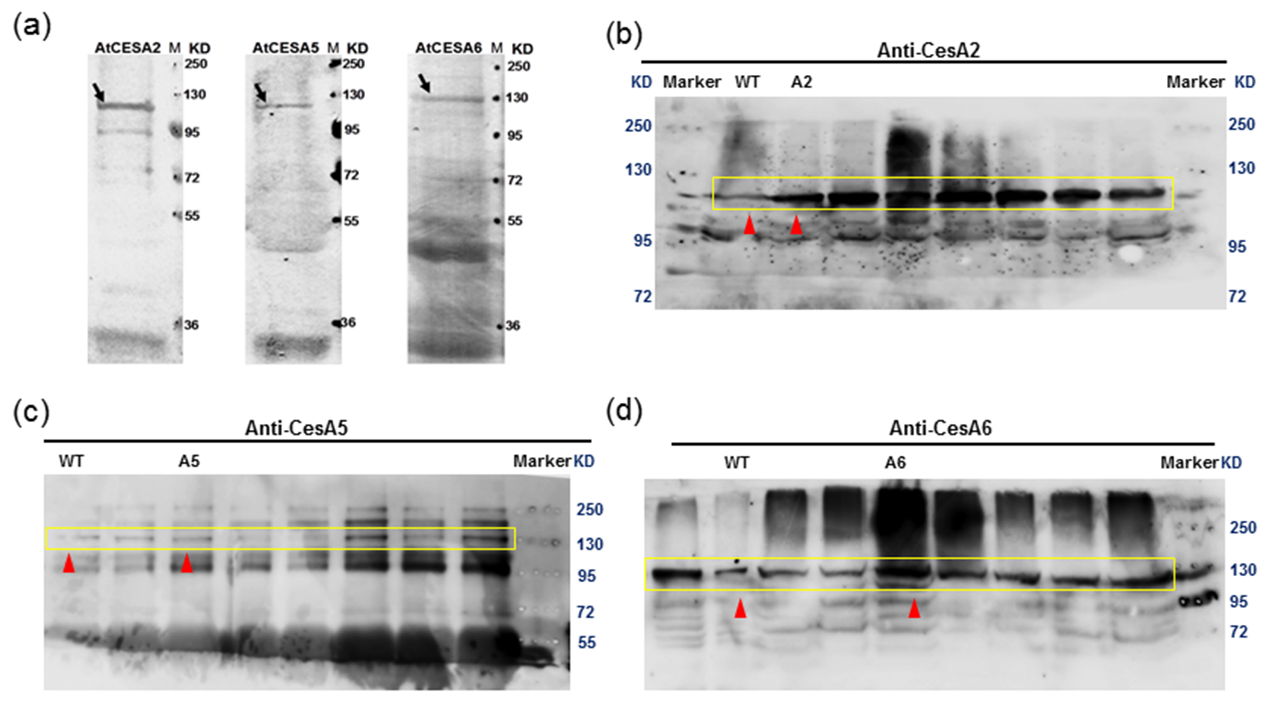


**Figure S2.** Analyses of AtCesA2, -5 and -6 protein levels in *Arabidopsis* seedlings. (a) Western blot detection of AtCesA2, -5 and -6 antibody specificity on membrane protein in WT seedlings. (b-d) Western blot analyses of AtCesA2, -5 and -6 proteins of D9 seedlings as the original images of Figure 1a*.*

**
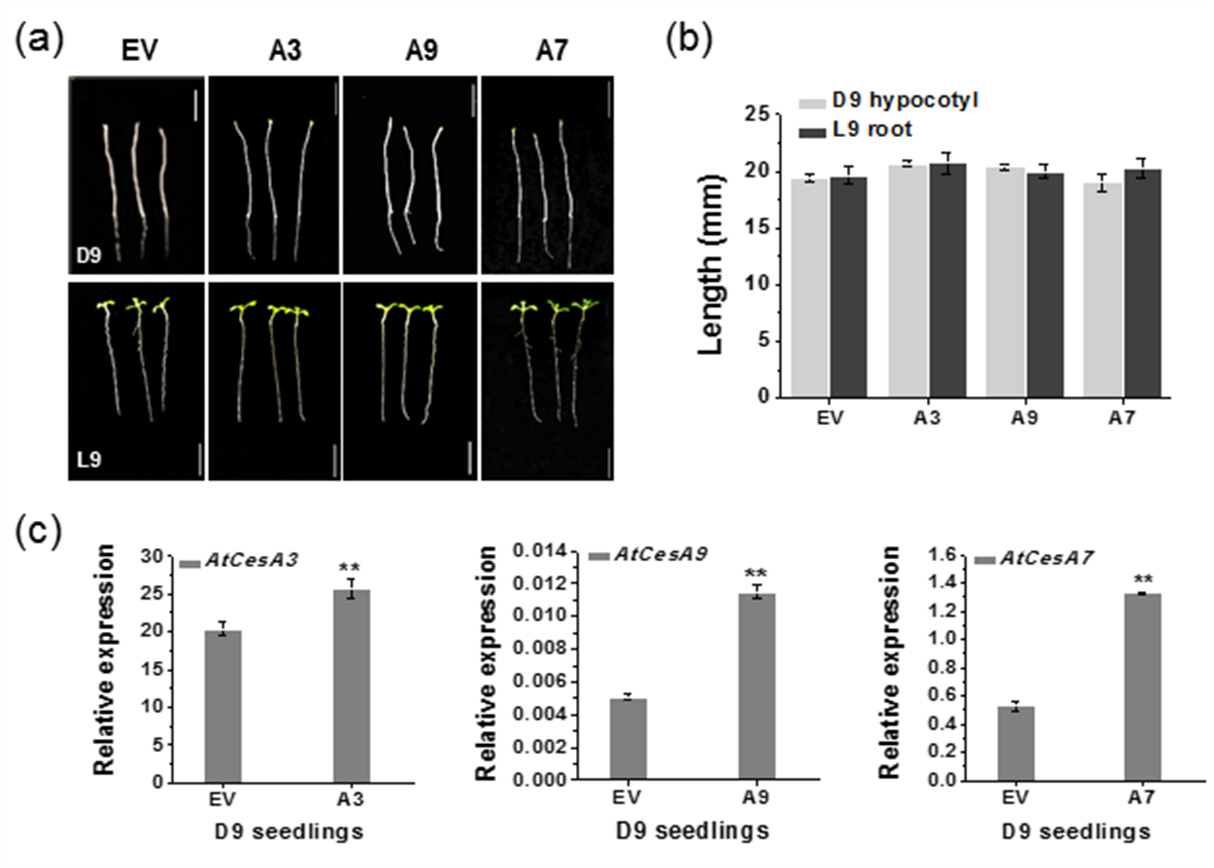
**

**Figure S3.** Observations of seedlings in other *CesA* genes over-expression lines. (a) The homozygous seedlings of EV and transgenic plants (A3, A9, A7) that over-express *CesA3*, *CesA9* and *CesA7* genes, respectively. Scale bars, 5 mm. (b) Hypocotyl and root lengths from seedlings as shown in (a). Bars indicated means ± SD (n=3 biological replicates), at least 30 plants were measured for each replicate; *P ≥* 0.05 by Student’s t-test. (c) Q-PCR analyses of A3, A9 and A7 D9 seedlings as shown in (a). *GAPDH* was used as the internal control and the expression value of *GAPDH* was defined as 100; Bars indicated means ± SD (n=3 biological replicates); ** *P* < 0.01 by Student’s t-test.

**
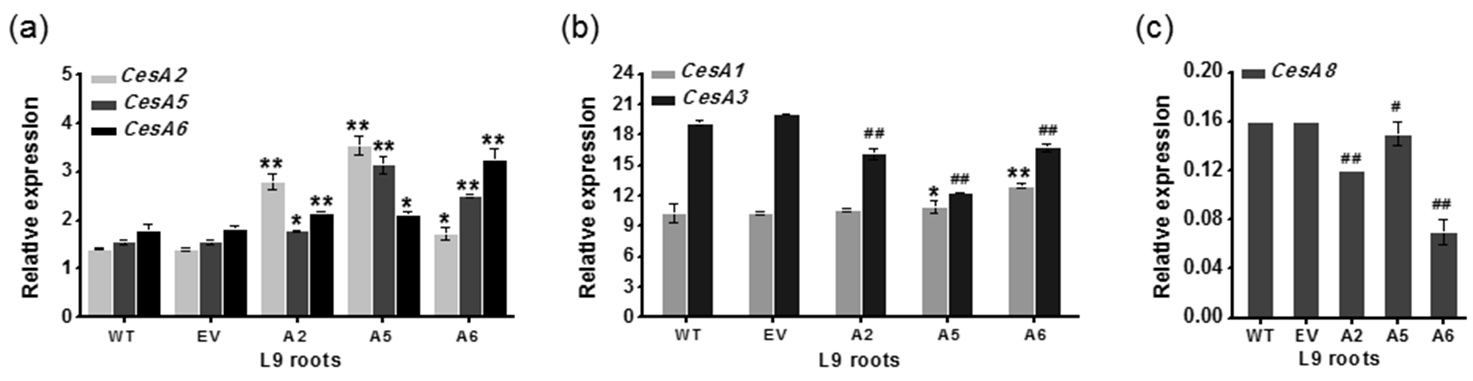
**

**Figure S4.** Q-PCR analyses of *CesA* genes in L9 roots of three *CesA6-*like genes over-expressing lines. *CesA2*, *CesA5*, or *CesA6* genes (a), *CesA1*, or *CesA3* genes (b) and *CesA8* gene (c). *GAPDH* was used as the internal control and the expression value of *GAPDH* was defined as 100; Bars indicated means ± SD (n=3 biological replicates); Student’s t-tests was performed between WT and transgenic plants as * *P* < 0.05 and ** *P* < 0.01 for increase, or ^#^ *P* < 0.05 and ^##^ *P* < 0.01 for decrease.


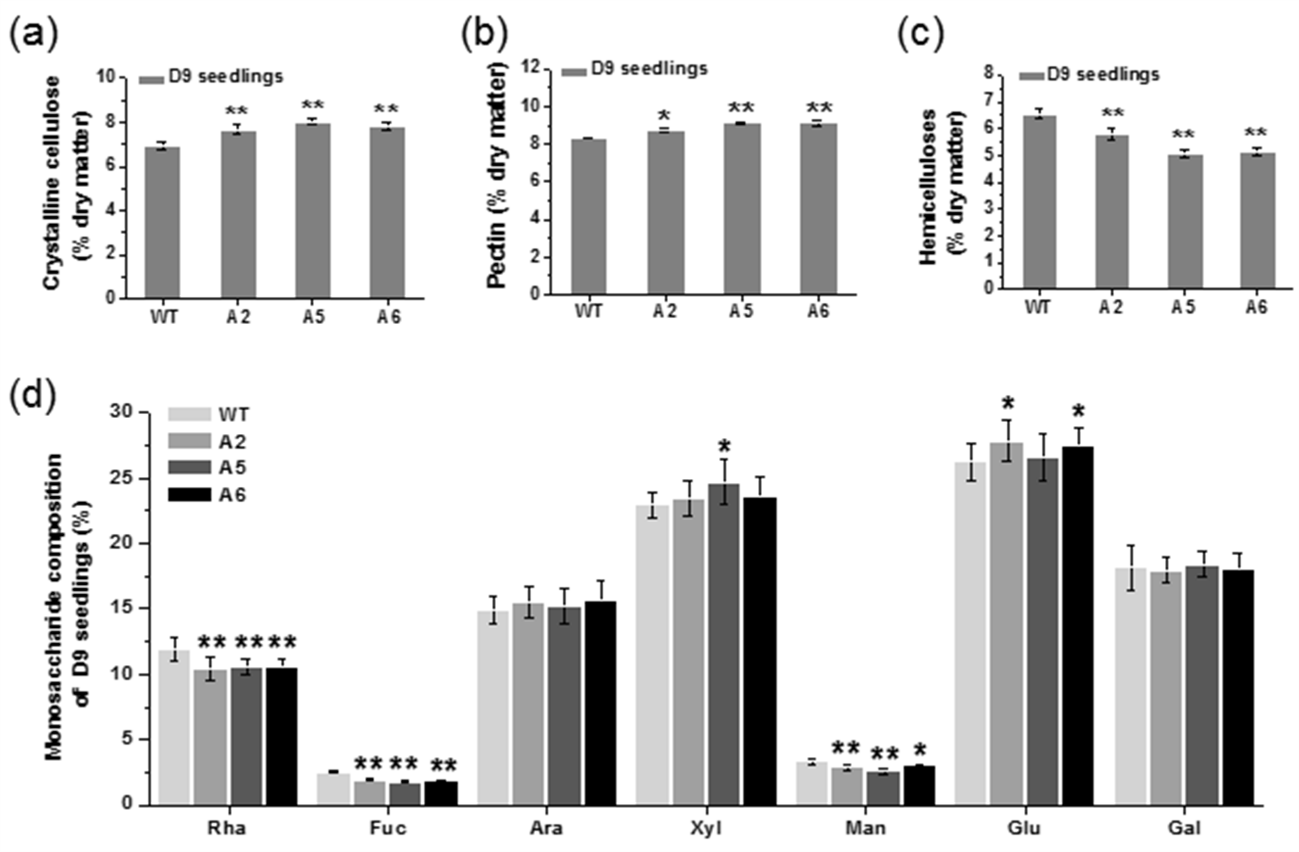


**Figure S5.** Cell wall compositions of D9 seedlings. (a-c) Relative cell wall compositions analyses of cellulose (a), pectin (b) and hemicelluloses (c). (d) Monosaccharide composition analyses of total wall polysaccharides by gas chromatography-mass spectrometer (GC-MS); Rha, rhamnose; Fuc, fucose; Ara, arabinose; Xyl, xylose; Man, mannose; Glu, glucose; Gal, galactose; Bars indicated means ± SD (n=3 biological replicates); * *P* < 0.05 and ** *P* < 0.01 by Student’s t-test.

**
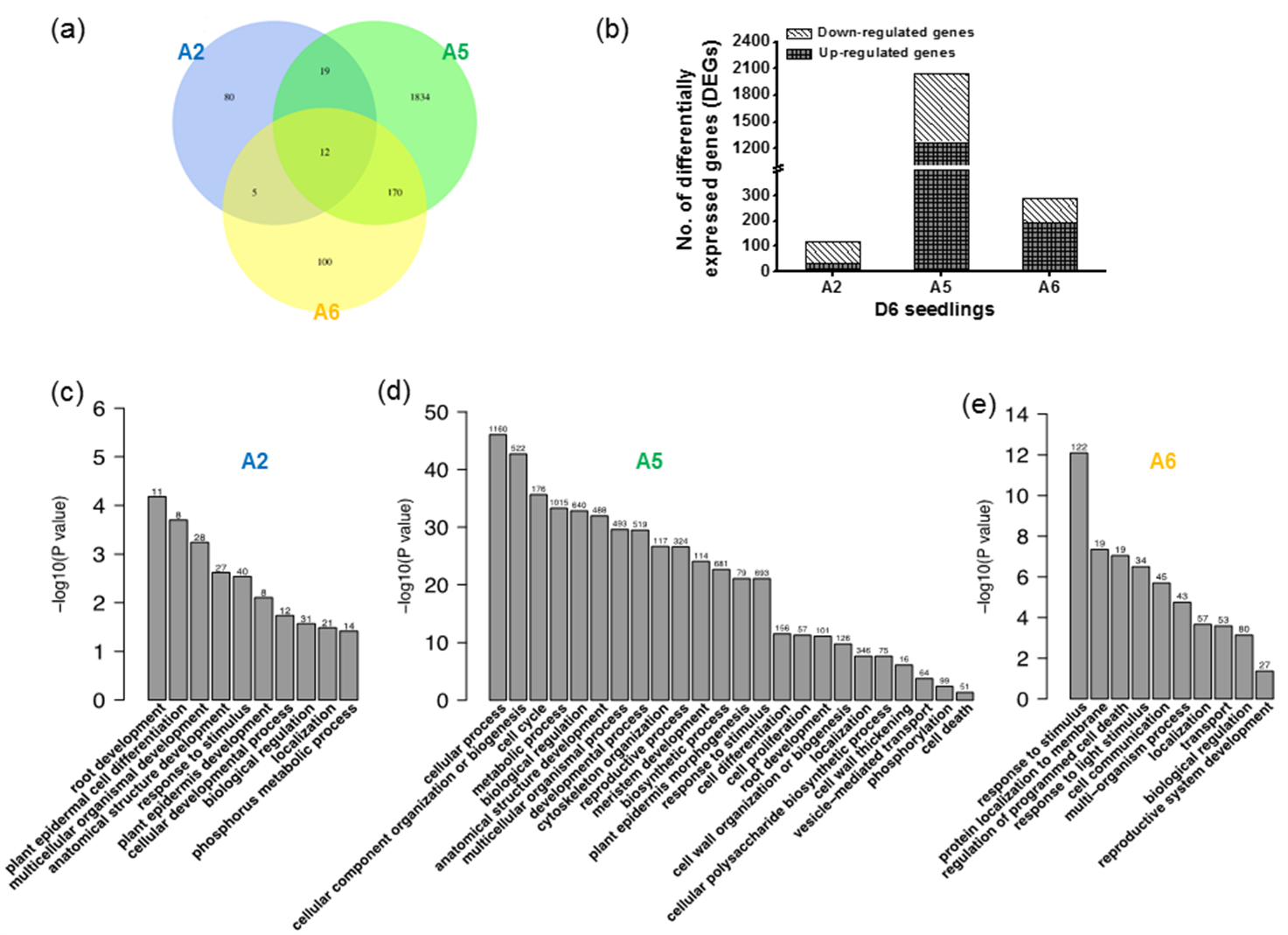
**

**Figure S6.** Altered expression of genes associated with cell growth by RNA sequencing. (a) Differentially expressed genes (DEGs) were identified by applying statistical tests (*P* < 0.001) for the genes between D6 transgenic (A2, A5 and A6) and WT seedlings. Blue, green and yellow represent *CesA2*, *CesA5* and *CesA6* over-expressing seedlings, respectively; Two biological replicates for each sample. (b) The number of up-regulated and down-regulated genes for each over-expression line as shown in (a). (c-e) Gene Ontology-Biological Process terms (GO-BP terms) of all DEGs in (a); A2 (c), A5 (d) and A6 (e). The enrichment analyses of GO-BP terms relative to its expectation were performed using a weighted method in combination with Fisher’s exact test.


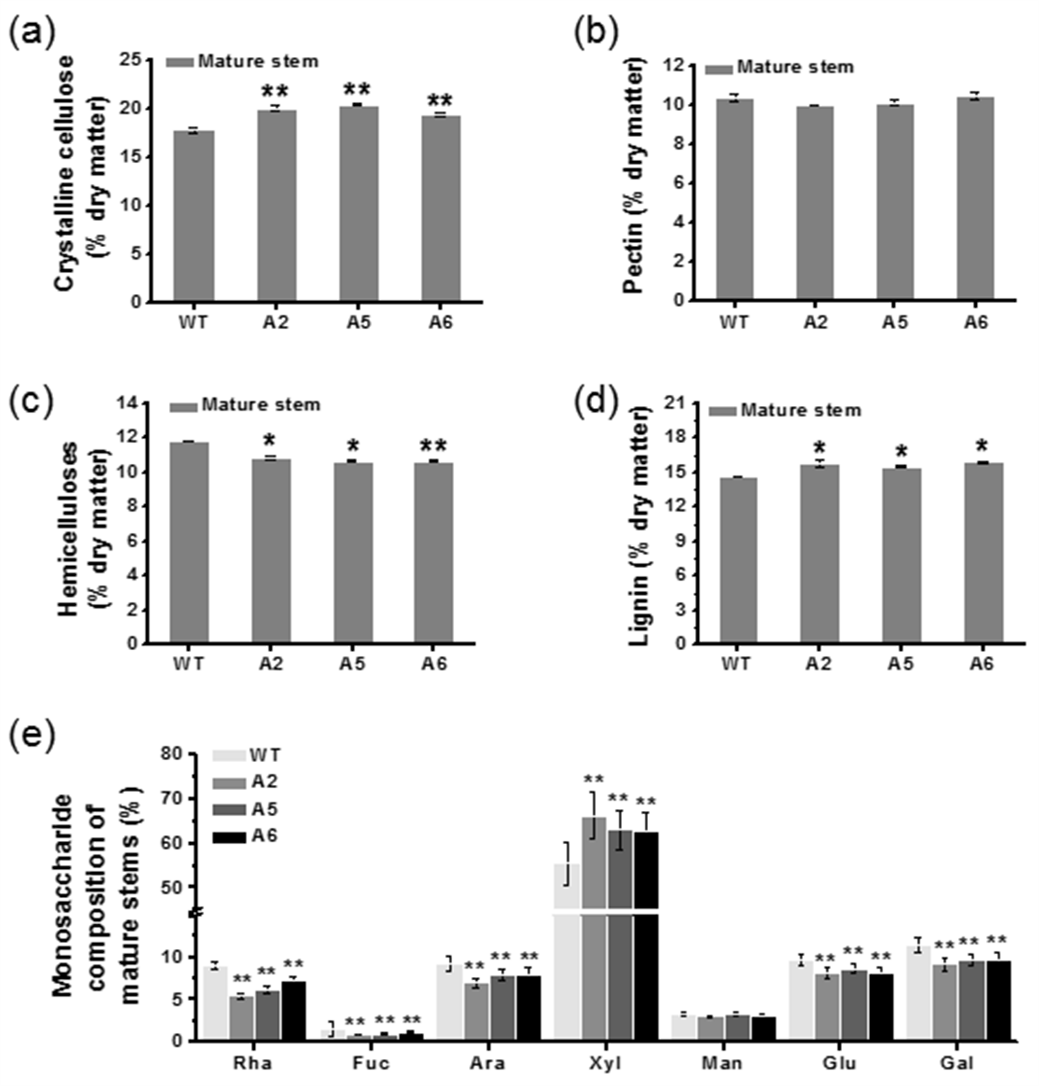


**Figure S7.** Cell wall compositions of seven-week-old inflorescence stems of mature plants. (a-d) Relative cell wall compositions analyses of cellulose (a), pectin (b), hemicelluloses (c) and lignin (d). (e) Monosaccharide composition analyses of total wall polysaccharides by GC-MS. Rha, rhamnose; Fuc, fucose; Ara, arabinose; Xyl, xylose; Man, mannose; Glu, glucose; Gal, galactose; Bars indicated means ± SD (n=3 biological replicates); * *P* < 0.05 and ** *P* < 0.01 by Student’s t-test.


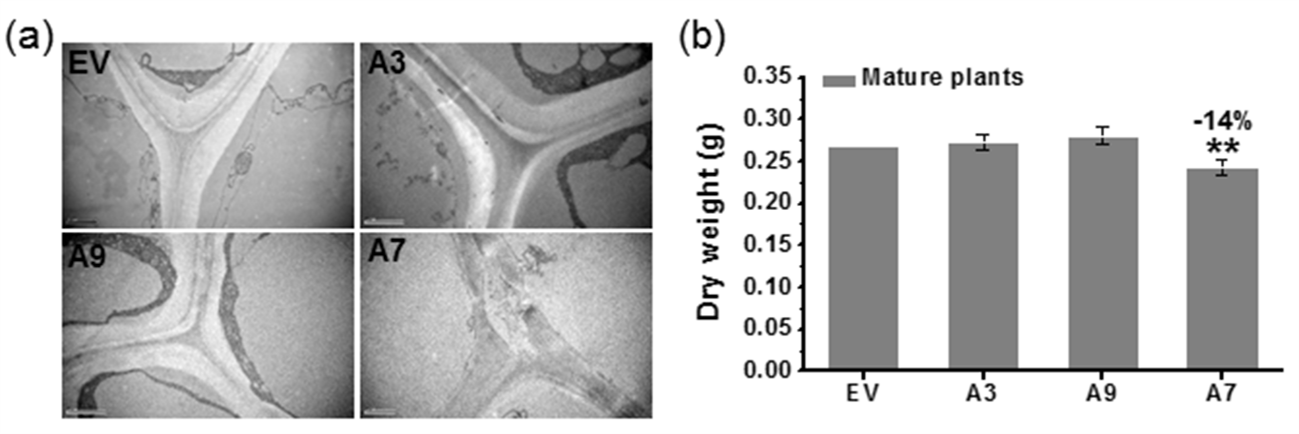


**Figure S8.** Observations of plants in other *CesA* over-expression lines. (a) Cell wall observations in xf tissues by TEM. Scale bars, 1 μm. (b) Dry weight of seven-week-old inflorescence stems of mature plants. Bars indicated means ± SD (n=3 biological replicates), and at least 30 plants were measured for each replicate; Student’s t-test as ** *P* < 0.01 between EV and over-expression lines.

**Supplementary tables**

**Table S1.** Q-PCR primers.

| **Genes** | **Primer name** | **Primer sequence (5’-3’)** | **TM (°C)** | **Length (bp)** | |
| --- | --- | --- | --- | --- | --- |
|  |  |  |  | **DNA** | **cDNA** |
| ***AtCesA1*** | **QA1-F** | AAGTGCTGCTATGTCCAGTTCCC | 58 | 401 | 295 |
|  | **QA1-R** | TGTTGATGCCTCTCCTCTTTTCG |  |  |  |
| ***AtCesA3*** | **QA3-F** | CCCTATCACCTCCATTCCTCTTCT | 58 | 334 | 179 |
|  | **QA3-R** | CGTCTATGCCTACGCCACTCC |  |  |  |
| ***AtCesA6*** | **QA6-F** | ACAGCACAGAAAGTGCCTGAG | 58 | 448 | 251 |
|  | **QA6-R** | GGAGCATTTGATAGAACCCCA |  |  |  |
| ***AtCesA2*** | **QA2-F** | TCGTCCCTGAGATAAGCAACTAC | 58 | 231 | 147 |
|  | **QA2-R** | CCCCTCCGATTACCCAAAA |  |  |  |
| ***AtCesA5*** | **QA5-F** | GATGCAATGGGGTAAAGTAGGG | 58 | 233 | 233 |
|  | **QA5-R** | TGATGAGTAGTGTGGTTGGAGGG |  |  |  |
| ***AtCesA9*** | **QA9-F** | GGAGGGAGACTCATTGCTGG | 58 | 675 | 255 |
|  | **QA9-R** | TGTATCGGGTTCCGCACTG |  |  |  |
| ***AtCesA4*** | **QA4-F** | ATTCTGGGTGATTGGCGG | 58 | 190 | 190 |
|  | **QA4-R** | AATAATGAGAGTTGTCGGAGGG |  |  |  |
| ***AtCesA7*** | **QA7-F** | TTCTTGCCTACTGTATCCTTCC | 58 | 231 | 152 |
|  | **QA7-R** | GCTAACTCCGCTCCATCTCA |  |  |  |
| ***AtCesA8*** | **QA8-F** | CATCCCAACGCTATCAAACCTA | 58 | 152 | 152 |
|  | **QA8-R** | CTGAGACACCTCCAATAACCCA |  |  |  |
| ***AtGAPDH*** | **QGAPDH-F** | GCAACATACGACGAAATCAAGAA | 58 | 398 | 217 |
|  | **QGAPDH-R** | CGACACGAGAACTGTAACCCC |  |  |  |

**Table S2.** Primers for over-expression vector construction.

| **Genes** | **Primer name** | **Primer sequence (5’-3’)** | **Restriction Enzyme cutting site** | **TM (°C)** | **Length (bp)** |
| --- | --- | --- | --- | --- | --- |
| ***AtCesA3*** | **A3-F** | GCTCTAGAATGGAATCCGAAGGAGAAACC | *XbaI* | 60 | 3329 |
|  | **A3-R** | AACTGCAGCACCAAGACAGAAGAACGAACAG | *PstI* |  |  |
| ***AtCesA6*** | **A6-F** | AAAGAGCTCATGAACACCGGTGGTCGG | *SacI* | 58 | 3255 |
|  | **A6-R** | AAATCTAGATCACAAGCAGTCTAAACCACAG | *XbaI* |  |  |
| ***AtCesA2*** | **A2-F** | GCTCTAGAATGAATACTGGTGGTCGGCTCAT | *XbaI* | 58 | 3255 |
|  | **A2-R** | AACTGCAGTTAGTTTCCACAATTCAGACCACAGA | *PstI* |  |  |
| ***AtCesA5*** | **A5-F** | GCTCTAGAATGAATACTGGTGGTCGGCTCATC | *XbaI* | 58 | 3210 |
|  | **A5-R** | AACTGCAGTCAAAGGCAGTCCAAGCCACATAT | *PstI* |  |  |
| ***AtCesA9*** | **A9-F** | GCTCTAGAATGAACACTGGAGGGAGACTC | *XbaI* | 56 | 3268 |
|  | **A9-R** | AACTGCAGCTCACTTTAAACAGTCAAGACCAC | *PstI* |  |  |
| ***AtCesA7*** | **A7-F** | GGGGTACCATGGAAGCTAGCGCCGGTC | *KpnI* | 60 | 3081 |
|  | **A7-R** | ACGCGTCGACTCAGCAGTTGATGCCACACTTG | *SalI* |  |  |
